# Supplementary material for: Empirical methods for controlling false positives and estimating confidence in ChIP-Seq peaks
Source: BMC Bioinformatics. 2008 Dec 5;9:523. doi: 10.1186/1471-2105-9-523 (PMC2628906; doi:10.1186/1471-2105-9-523)
Supplement: Additional File 1 — A variety of html documents from the USeq web site detailing the available applications, their best usage, output file type descriptions, command line menus, etc. [file 1471-2105-9-523-S1.zip › USeqUserGuides/JavaDoc.zip!/JavaDoc/gata/aligner/AlignParams.html]

AlignParams


|  |  |  |
| --- | --- | --- |
| **Overview** \| **Package** \| **Class** \| **Use** \| **Tree** \| **Deprecated** \| **Index** \| **Help** | |  |
| **PREV CLASS**   **NEXT CLASS** | **FRAMES**    **NO FRAMES**     **All Classes** |  |
| SUMMARY: NESTED \| FIELD \| CONSTR \| METHOD | DETAIL: FIELD \| CONSTR \| METHOD |  |


---


## gata.aligner Class AlignParams

```
java.lang.Object
  gata.aligner.AlignParams
```

**All Implemented Interfaces:**: java.io.Serializable

---

``` public class AlignParams extends java.lang.Object implements java.io.Serializable ```

**Author:**
:   nix
    A class to represent alignment parameters entered for a particular alignment. This is
    saved and made availible to GATAPlotter.

**See Also:**: Serialized Form

---

**Constructor Summary**

`AlignParams()`

`AlignParams(AlignerPreferences ap)`


| **Method Summary** | |
| --- | --- |
| `java.lang.String` | `getBaseName()` |
| `java.lang.String` | `getBl2seq()` |
| `java.lang.String` | `getCompSeqFile()` |
| `java.lang.String[]` | `getDNAFILES()` |
| `boolean` | `getDUST()` |
| `double` | `getEFF_M()` |
| `double` | `getEFF_N()` |
| `int` | `getGAP_CREATE()` |
| `int` | `getGAP_EXT()` |
| `double` | `getH()` |
| `double` | `getK()` |
| `double` | `getLAMBDA()` |
| `int` | `getLENGTH_COMPSEQ()` |
| `int` | `getLENGTH_REFSEQ()` |
| `int` | `getMATCH()` |
| `int` | `getMAX_SCORE()` |
| `int` | `getMIN_SCORE()` |
| `int` | `getMISMATCH()` |
| `java.lang.String` | `getNAME_COMPSEQ()` |
| `java.lang.String` | `getNAME_REFSEQ()` |
| `java.lang.String` | `getPathToResults()` |
| `java.lang.String` | `getRefSeqFile()` |
| `int` | `getSTART_INDEX_COMPSEQ()` |
| `int` | `getSTART_INDEX_REFSEQ()` |
| `int` | `getWIN_SIZE()` |
| `boolean` | `isCompVisible()` |
| `boolean` | `isEXTRACT()` |
| `void` | `setCompVisible(boolean compVisible)` |
| `void` | `setEFF_M(double d)` |
| `void` | `setEFF_N(double d)` |
| `void` | `setEXTRACT(boolean extract)` |
| `void` | `setH(double d)` |
| `void` | `setK(double d)` |
| `void` | `setLAMBDA(double d)` |
| `void` | `setLENGTH_COMPSEQ(int l)` |
| `void` | `setLENGTH_REFSEQ(int l)` |
| `void` | `setNAME_COMPSEQ(java.lang.String s)` |
| `void` | `setNAME_REFSEQ(java.lang.String s)` |
| `void` | `setSTART_INDEX_COMPSEQ(int start_index_compseq)` |
| `void` | `setSTART_INDEX_REFSEQ(int start_index_refseq)` |
| `java.lang.String` | `toHTMLString()` |

**Methods inherited from class java.lang.Object**

`equals, getClass, hashCode, notify, notifyAll, toString, wait, wait, wait`

**Constructor Detail**

### AlignParams

```
public AlignParams()
```

---


### AlignParams

```
public AlignParams(AlignerPreferences ap)
```


**Method Detail**

### getWIN\_SIZE

```
public int getWIN_SIZE()
```

---


### getMATCH

```
public int getMATCH()
```

---


### getMISMATCH

```
public int getMISMATCH()
```

---


### getGAP\_CREATE

```
public int getGAP_CREATE()
```

---


### getGAP\_EXT

```
public int getGAP_EXT()
```

---


### getMIN\_SCORE

```
public int getMIN_SCORE()
```

---


### getMAX\_SCORE

```
public int getMAX_SCORE()
```

---


### getSTART\_INDEX\_REFSEQ

```
public int getSTART_INDEX_REFSEQ()
```

---


### getSTART\_INDEX\_COMPSEQ

```
public int getSTART_INDEX_COMPSEQ()
```

---


### getLENGTH\_REFSEQ

```
public int getLENGTH_REFSEQ()
```

---


### getLENGTH\_COMPSEQ

```
public int getLENGTH_COMPSEQ()
```

---


### getDNAFILES

```
public java.lang.String[] getDNAFILES()
```

---


### getNAME\_REFSEQ

```
public java.lang.String getNAME_REFSEQ()
```

---


### getNAME\_COMPSEQ

```
public java.lang.String getNAME_COMPSEQ()
```

---


### getPathToResults

```
public java.lang.String getPathToResults()
```

---


### getDUST

```
public boolean getDUST()
```

---


### setLENGTH\_REFSEQ

```
public void setLENGTH_REFSEQ(int l)
```

---


### setLENGTH\_COMPSEQ

```
public void setLENGTH_COMPSEQ(int l)
```

---


### setNAME\_REFSEQ

```
public void setNAME_REFSEQ(java.lang.String s)
```

---


### setNAME\_COMPSEQ

```
public void setNAME_COMPSEQ(java.lang.String s)
```

---


### toHTMLString

```
public java.lang.String toHTMLString()
```

---


### getEFF\_M

```
public double getEFF_M()
```

---


### getEFF\_N

```
public double getEFF_N()
```

---


### getH

```
public double getH()
```

---


### getK

```
public double getK()
```

---


### getLAMBDA

```
public double getLAMBDA()
```

---


### setEFF\_M

```
public void setEFF_M(double d)
```

---


### setEFF\_N

```
public void setEFF_N(double d)
```

---


### setH

```
public void setH(double d)
```

---


### setK

```
public void setK(double d)
```

---


### setLAMBDA

```
public void setLAMBDA(double d)
```

---


### getBaseName

```
public java.lang.String getBaseName()
```

---


### getBl2seq

```
public java.lang.String getBl2seq()
```

---


### getCompSeqFile

```
public java.lang.String getCompSeqFile()
```

---


### getRefSeqFile

```
public java.lang.String getRefSeqFile()
```

---


### setSTART\_INDEX\_COMPSEQ

```
public void setSTART_INDEX_COMPSEQ(int start_index_compseq)
```

---


### setSTART\_INDEX\_REFSEQ

```
public void setSTART_INDEX_REFSEQ(int start_index_refseq)
```

---


### isEXTRACT

```
public boolean isEXTRACT()
```

---


### setEXTRACT

```
public void setEXTRACT(boolean extract)
```

---


### isCompVisible

```
public boolean isCompVisible()
```

---


### setCompVisible

```
public void setCompVisible(boolean compVisible)
```


---


|  |  |  |
| --- | --- | --- |
| **Overview** \| **Package** \| **Class** \| **Use** \| **Tree** \| **Deprecated** \| **Index** \| **Help** | |  |
| **PREV CLASS**   **NEXT CLASS** | **FRAMES**    **NO FRAMES**     **All Classes** |  |
| SUMMARY: NESTED \| FIELD \| CONSTR \| METHOD | DETAIL: FIELD \| CONSTR \| METHOD |  |


---
